# Suicidal Behavior and Its Association With Psychological Distress, Coping Mechanisms, and Resilience: A Cross-Sectional Study

**DOI:** 10.7759/cureus.60322

**Published:** 2024-05-15

**Authors:** Mayura Vimalanathane, Pallavi Abhilasha, Amritha Prasad, Arul Saravanan Ramachandran, Karthick Subramanian

**Affiliations:** 1 Department of Psychiatry, Mahatma Gandhi Medical College and Research Institute, Sri Balaji Vidyapeeth (Deemed-to-be University), Puducherry, IND; 2 Department of Psychiatry, Christian Medical College, Ludhiana, IND; 3 Department of Psychiatry, Sree Gokulam Medical College Hospital and Research Foundation, Trivandrum, IND; 4 Department of Psychiatry, SRM (Sri Ramaswamy Memorial) Medical College Hospital & Research Institute, SRM (Sri Ramaswamy Memorial) Institute of Science and Technology, Chennai, IND

**Keywords:** india, suicide, stressful life events, risk factors, protective factors

## Abstract

Introduction

Recent literature reveals that psychological factors such as resilience and coping mechanisms can act as buffers against suicide risk. Indian literature on the interplay between psychological risk and protective factors of suicidal behavior is scarce.

Methods

A cross-sectional descriptive study was done among suicide attempters in a tertiary care hospital in Southern India. A semi-structured proforma was used to obtain sociodemographic data and suicide attempt characteristics. Suicide intent, lethality, stressful life events, perceived stress, subjective distress, coping strategies, and resilience were recorded using standard rating scales. Inferential analyses were carried out with p≤ 0.05 set as statistical significance.

Results

Pesticide poisoning (46.7%) was the most common mode of suicide attempt. Significant gender differences emerged in the mode of suicide attempt, coping strategies, and resilience. Depression (48.7%) was the most common psychiatric comorbidity. Increased perceived stress was associated with the presence of psychiatric comorbidity, past history of suicide attempts, and high-intent suicide attempts. Maladaptive coping strategies were associated with substance abuse and a history of past suicide attempts. Low resilience levels were associated with hanging attempts, psychiatric or substance use disorder comorbidity, past history of suicide attempts, high-intent suicide attempts, and less lethal suicide attempts.

Conclusion

Perceived stress levels, coping strategies, and resilience have significant relationships with suicidal behavior and act as avenues for suicide prevention efforts.

## Introduction

Suicide remains a significant global public health concern, more pronounced (almost 77% of global suicides) in the low- and middle-income nations [1]. In India, every year, more than one lakh people succumb to suicide, reflecting an approximate annual rate of 12.4 per lakh population [2]. India’s annual proportional contribution to the global suicide death rate among men and women is increasing, which is quite alarming [3-5]. The recent coronavirus disease 2019 (COVID-19) pandemic has accentuated the rates of suicide among the general population [6]. A dynamic exploration and updating of information on the risk factors and protective factors of suicidal behavior will help mitigate the risk in susceptible individuals and plan for timely interventions.

Risk factors for suicide

Various risk factors for suicidal behavior have been identified among the Indian population: age groups of 18-30 years, being married, family conflict, physical illness, drug abuse, financial difficulties, etc. [2] Apart from such factors, the literature consistently reveals that individual susceptibility to suicidal behavior can be governed by numerous factors including personality characteristics, cognitive schemas, and the social milieu where the individual is placed [7-9]. 

Psychological risk factors

Personality-Related Factors

The literature reveals various individual-related psychological risk factors for suicide: cluster B personality traits, trait impulsivity, aggression, perfectionism, conduct disorder, antisocial behavior, and decreased resilience, among others [7-12]. Temperamental characteristics such as novelty seeking, and harm avoidance were noted to be linked to suicidal behavior [7]. While the above can be grouped as distal psychological risk factors, the proximal psychological risk factors are mainly depression, substance abuse, bipolar disorder, and schizophrenia [7,13]. Hopelessness remains a significant predictor of impending suicidal behavior and of follow-up suicide attempts [12,14]. 

Cognitive Factors

Constructs such as cognitive rigidity/inflexibility, thought suppression, rumination, pain insensitivity, and implicit associative thoughts about death are reported to be strongly associated with suicidal behavior [9]. Low levels of resilience were more frequently noted among suicide attempters than non-attempters [15]. Another recent study during the COVID-19 pandemic identified that high resilience levels reduced the negative impact of the pandemic and the associated suicide risk [16]. On the contrary, enhanced resilience levels act as a protective factor against suicide [17]. In addition, increased levels of perceived stress and perceived burdensomeness moderate the protective effects of resilience and optimism on suicidal behavior [18,19]. 

Social Factors

It is well known that stressful life events can trigger new-onset suicidal ideation among susceptible individuals [20]. A significant proportion of suicides in India happen secondary to stressful life events, including family conflict, financial hardships, unemployment, reduced economic growth, relationship issues, chronic pain, and illness-related issues [1,21-23]. Social isolation during the COVID-19 pandemic has been highlighted to be associated with increased suicidal behavior [24]. The consequent higher levels of perceived distress were associated with “intent to die” by suicide [25,26]. In addition, increased levels of perceived stress can act as a marker for impending suicidal ideation secondary to stressful life events (SLEs) [27]. On the contrary, high levels of perceived interpersonal support were associated with a diminished risk of suicide [28].

Psychological protective factors

Coping Strategies

Deficits in problem-solving and coping with such adversities were associated with suicidal behavior [12,29,30]. Investigations into coping mechanisms and suicidal behavior reveal that individuals with a recent history of suicide attempts avoided active coping and positive reinterpretation and employed more maladaptive strategies such as avoidant coping, denial, disengagement, and substance abuse among others [31,32]. Less healthy coping strategies among suicide attempters were associated with reduced quality of life [33,34]. On the contrary, seeking social support, accepting responsibility, and problem-solving approaches were associated with reduced chances of suicide attempts [35,36].

Resilience

Resilience is a psychobiological construct defined as the “human capacity to adapt swiftly and successfully to stressful/traumatic events and manage to revert to a positive state [37].” Recent studies reveal that high levels of optimism and resilience, especially among young adults, were associated with a reduced risk of suicidal behavior [12,38]. Certain internal attributes (such as positive coping strategies, psychological capital, having meaning/purpose in life, and a sense of responsibility) and external mechanisms (such as enhanced social support/positive relationships and an inclusive environment) have been identified as crucial contributors for resilience towards suicidal ideations [39-41]. Among young adults, positive thinking and perceived social support have been found to mediate the beneficial effects of resilience on suicide risk [42].

The complex interplay among sociocultural, socioeconomic, and ethnic factors highlights the need for a better understanding of risk factors for suicide in developing countries like India [10,43]. The southern states of India have reported a greater suicide rate when compared to other regions [2]. The recent NCRB (2022) data reveals that urban domiciles, especially in metropolitan areas like Chennai, are associated with an increased risk of suicide and act like “suicide clusters [2,44].” Though numerous Indian studies on psychological risk factors for suicide are present [31,45-49], studies on the interactions between psychological risk factors and protective factors, such as resilience, among suicide attempters are rare in the Indian context [33,35,36,50]. The present study aimed to assess the association between current suicidal behavior, psychological distress, coping strategies, and resilience among suicide attempters.

## Materials and methods

Study setting, design, and participants

The cross-sectional descriptive study was conducted in a tertiary care teaching hospital (SRM Medical College Hospital & Research Centre, Chennai, India) among suicide attempters. Patients aged 18 years and older who attempted suicide in the past month were included in the study through the purposive sampling method. Patients with accidental suicide attempts, those who were unwilling to participate, and those who were medically unstable or experienced acute withdrawal states or delirium were excluded from the study.

Study definitions

A suicide attempt is defined as “a non-fatal self-directed potentially injurious behavior with an intent to die [51].” A suicide attempter is any individual with a history of suicide attempts in the past month.

Study instruments

Sociodemographic Proforma

A semi-structured proforma was used to record the patient’s sociodemographic characteristics (age, sex, residence, religion, type of family, occupation, education, socioeconomic status, marital status), suicide attempt characteristics (past attempt history, mode of suicide attempt, suicide intent, relation to intoxication, duration of suicidal ideas), family history of suicide, and clinical characteristics (presence of psychiatric illness and comorbid substance use).

Beck Suicide Intent Scale

Beck’s suicide intent scale contains 20 items, each scoring from 1 to 3 points. The total score of 15-19 was recorded as low intent, the score of 20-28 was recorded as medium intent, and the score of 29 and above was recorded as high intent. The scale is used to screen those with high suicidal intent and explore the factors associated with intent [52].

Presumptive Stressful Life Events Scale (PSLES)

The PSLES consists of 51 life events (with weighted scores ranging from 0 to 100) commonly experienced by the average Indian adult population in the past year. Scale items were further classified into (a) desirable, undesirable, or ambiguous and (b) personal or impersonal (not dependent on the individual action) [53]. Each type of SLE is given a weightage score, and the total life event severity score is obtained by summing the weightage scores of SLEs encountered by the patients prior to the suicide attempt.

Perceived Stress Scale (PSS)

The PSS measures the degree of self-appraisal of stressful life events in the past month [54]. The scale has 10 items, measured on a 5-point Likert scale (0-never, 1-Almost Never, 2-Sometimes, 3-Fairly Often, 4-Very Often). Four items (items 4, 5, 7, & 8) are reverse-coded and the total score is obtained by summing all scores. Higher scores reveal more significant levels of distress. The PSS scale has demonstrated good validity as reflected by Cronbach’s alpha of 0.78.

General Health Questionnaire (GHQ-12)

GHQ-12 measures psychological distress in a quick, reliable, and sensitive short format [55]. The 12 items are graded on a 4-point Likert scale (0-3) and scores ranging between 0 and 36. A score >15 is evidence of distress, and a score >20 suggests severe problems and psychological distress. Cronbach’s alpha for GHQ12 is 0.9.

COPE Inventory Scale

The COPE Inventory assesses an individual’s dispositional and situational coping strategies. It comprises 15 subscales, each rated on a 4-point Likert scale (1=‘‘usually do not do this at all” to 4=‘‘usually do this a lot”), which assesses various coping strategies. The 15 subscales are categorized into five domains: problem-focused coping, emotion-focused coping, less useful coping, substance use, and humor. Cronbach’s alpha for the 15 scales ranged from 0.37 to 0.93. The average alpha was 0.79 [56].

Connor-Davidson Resilience Scale (CD-RISC)

The CD-RISC contains 25 items, each with a 5-point Likert response: not true at all (0), rarely true (1), sometimes true (2), often true (3), and true nearly all of the time (4). CD-RISC measures the subjective feeling over the past month [57]. The total score ranges between 0 and 100, with higher scores reflecting greater resilience. Cronbach’s alpha for the Connor-Davidson Resilience scale is 0.82 to 0.92.

Risk Rescue Rating Scale (RRRS)

The RRRS was administered to evaluate the lethality of the current suicide attempt [58]. The scale comprises 10 items grouped into two subscales: one assessing the risk factors (method of attempt, impaired consciousness, toxicity, reversibility, and treatment required) and the other assessing the rescue factors (location, the person initiating a rescue, probability of discovery, accessibility for rescue, and delay until discovery). Both the risk score and rescue score ranged between 1 and 5, with higher scores in the risk subscale indicating greater lethality and higher scores in the rescue subscale indicating less lethal/more rescuable suicide attempts. The risk/rescue ratio is calculated using the formula [Risk score/(Risk score + Rescue score)] × 100. There is no cut-off score of RRRS to define a fatal suicide attempt.

Study procedure

After study inclusion, the patient’s sociodemographic, suicidal attempt, and clinical characteristics are entered into a semi-structured proforma. Using standard validated scales, the psychological characteristics such as stressful life events, resilience, coping strategies, perceived stress, and psychological distress. The scales were translated to Tamil and back-translated to English using the help of two independent linguistic experts proficient in Tamil and English. The resultant forms were verified for content by one of the study investigators who was not involved in the data collection process (KS). The patients were encouraged to answer the self-rated instruments, and the primary investigator (MV) clarified their doubts during the process. Written informed consent was obtained from all the study participants, and the Institute Ethics Committee approved the study protocol (1130/IEC/2017).

Statistical analysis

The distribution of continuous variables was depicted using means and standard deviation, and that of categorical variables using frequency and percentages. Comparison of means of normally distributed continuous variables was performed using independent sample t-test and One-way ANOVA. The differences between categorical variables were computed using the chi-square test. Correlation analyses were done among the variables - psychological distress, coping strategies, resilience, and risk-rescue scores using Pearsons’s correlation analyses. Data analysis was done using IBM SPSS Statistics for Windows, Version 17 (Released 2008; IBM Corp., Armonk, New York, United States). Statistical significance was set at p ≤ 0.05.

## Results

Sociodemographic characteristics of suicide attempters

One hundred and fifty patients participated in the study. The majority of the patients were females (62.7%), Hindus (86.7%), homemakers (32%), belonged to the 2nd and 4th decades of life, hailed from low socioeconomic status (51.3%), completed up to high-school education (38.7%), and lived in semi-urban domicile (56.7%). Most patients were married (57.3%) and lived in nuclear families (87.3%), and 24% of patients reported a family history of suicide (Table 1).

**Table 1:** Sociodemographic characteristics of patients (N=150)

Variables	Frequency (percentage) or Mean ± SD
Age (in years)	28.56 ± 10.39
Gender	
Male	56 (37.3)
Female	94 (62.7)
Religion	
Hindu	130 (86.7)
Christian	12 (8)
Muslim	8(5.3)
Socioeconomic status	
Low	77 (51.3)
Middle	65 (43.3)
High	8 (5.4)
Education	
Illiterate	6 (4)
Primary	0
Middle	10 (6.7)
High	58 (38.7)
Higher	26 (17.2)
UG	40 (26.7)
PG	10 (6.7)
Occupation	
Housewife	48 (32)
Semiskilled	40 (26.7)
Skilled	14 (9.3)
Professional	13 (8.7)
Unemployed	7 (4.7)
Student	28 (18.7)
Residence	
Rural	52 (34.7)
Semiurban	85 (56.7)
Urban	13 (8.6)
Type of family	
Nuclear	131 (87.3)
Joint	19 (12.7)
Family h/o suicide	
Yes	36 (24)
No	114 (76)
Marital status	
Single	63 (42)
Married	86 (57.3)
Divorced	1 (0.7)

Gender differences among suicide attempters

Gender-based comparisons revealed that pesticide poisoning and tablet overdose were the most common modes of attempt among males and females, respectively (p=0.02). Substance use was exclusively noted only in males (p<0.001). Both male and female suicide attempters had a high frequency of medium-intent suicide attempts (p=0.02). The lethality of current suicide attempts did not differ between the two groups. Though psychological distress was similar between male and female suicide attempters, coping strategies and resilience differed between the two genders. While emotion-focused coping was more prominent among female attempters, substance-mediated coping was exclusively noted among male attempters (p<0.001). Also, female suicide attempters had lower resilience scores when compared to male suicide attempters (p=0.02) (Table 2).

**Table 2:** Gender differences among suicide attempters (N=150) *p<0.05, statistically significant CD-RISC: Connor Davidson Resilience Scale, GHQ: General Health Questionnaire, PSLES: Presumptive Stressful Life Events Scale.

S.No	Variable	Males (n=56) Frequency (percentage)	Females (n=94) Frequency (percentage)	Test characteristic, p-value
1	Age (in years)	30.64 ± 12.64	27.32 ± 8.61	t= 1.913 (0.06)
2	Mode of attempt			
	Pesticide poisoning	32 (57.1)	38 (40.4)	χ^2 ^= 11.320 (0.01)*
	Tablet overdose	10 (17.9)	41 (43.6)	
	Household poisons	7 (12.5)	10 (10.6)	
	Hanging	7 (12.5)	5 (5.4)	
3	Suicide intent			
	Low intent	11 (19.6%)	39 (41.5%)	U = 1992.0 (0.01)*
	Medium intent	32 (57.1%)	42 (44.7%)	
	High intent	13 (23.2%)	13 (13.8%)	
	Mean Rank of suicide intent (ordinal)	86.93	68.69	
4	Lethality of current suicide attempt			
	Risk score	7.27 ± 1.24	7.27 ± 1.32	t=0.009 (0.99)
	Rescue score	13.80 ± 1.80	14.04 ± 1.39	t=-0.909 (0.36)
	Risk-rescue score	30.77 ± 10.50	29.07 ± 9.04	t=1.044 (0.29)
5	Duration of suicidal ideas			
	Sudden Impulse	47 (83.9)	88 (93.6)	Fisher’s Exact = 5.318 (ns)
	1-4 Weeks	7 (12.5)	4 (4.2)	
	1-6 Months	2 (3.6)	1 (1.1)	
	7-12 Months	0 (0)	1 (1.1)	
6	Past Attempt			
	Yes	12 (21.4)	23 (24.5)	χ^2 ^= 0.181 (0.67)
	No	44 (78.6)	71 (75.5)	
7	Family h/o suicide			
	Yes	16 (28.6)	20 (21.3)	χ^2 ^= 1.024 (0.31)
	No	40 (71.4)	74 (78.7)	
8	Psychiatric comorbidity			
	Depression	28 (50)	45 (47.9)	Fisher’s Exact = 0.227 (ns)
	Schizophrenia	1 (1.8)	1 (1.1)	
	None	27 (48.2)	48 (51.1)	
9	Substance use comorbidity			
	Yes	35 (62.5)	0 (0)	Fisher’s Exact= 76.630 (<0.001)*
	No	21 (37.5)	94 (100)	
10	Psychological characteristics			
10.1	Psychological distress			
	PSLES total	163.25 ± 79.31	162.54 ± 84.86	t=0.051 (0.96)
	Perceived stress scale score	24.55 ± 6.04	23.57 ± 6.59	t=0.908 (0.37)
	GHQ scale score	11.39 ± 4.15	11.79 ± 5.29	t=-0.477 (0.63)
10.2	Coping strategies			
	Problem-focused coping	49.38 ± 6.88	51.35 ± 7.74	t=-1.576 (0.11)
	Emotion-focused coping	47.54 ± 6.19	52.34 ± 8.18	t=-3.794 (<0.001)*
	Less useful coping	32.21 ± 4.63	33.72 ± 4.90	t=-1.862 (0.06)
	Substance use coping	10.04 ± 5.49	0	t=10.67 (<0.001)*
	Humor	4.11 ± 0.59	4.33 ± 1.14	t=-1.123 (0.26)
10.3	Resilience			
	CD-RISC score	74.77 ± 11.15	69.77 ± 12.71	t=1.913 (0.02)*

Suicide attempt characteristics

The majority of suicide attempts were due to pesticide poisoning (46.7%), followed by tablet overdose (34%) and consumption of household poisons (11.3%). Most suicide attempts were impulsive in nature (90%), with medium intent (49.3%), and only a few numbers (11.3%) were attempted under intoxication. A history of past suicide attempt was noted among 23.3% of the sample (Table 3).

**Table 3:** Suicide attempt characteristics among suicide attempters (N=150)

Variable	Frequency (percentage) or Mean ± SD
Mode of current attempt	
Pesticide poisoning	70 (46.7)
Tablet overdose	51 (34)
Household poisons	17 (11.3)
Hanging	12 (8)
Suicide intent	
Low intent	50 (33.3)
Medium intent	74 (49.3)
High intent	26 (17.4)
Lethality of current suicide attempt	
Risk score	7.27 ± 1.29
Rescue score	13.95 ± 1.56
Risk-rescue score	29.71 ± 9.61
Under Intoxication	
Yes	17 (11.3)
No	133 (88.7)
Duration of suicidal ideas	
Sudden Impulse	135 (90)
1-4 Weeks	11 (7.3)
1-6 Months	3 (2)
7-12 Months	1 (0.7)
Past Attempt	
Yes	35 (23.3)
No	115 (76.7)

Clinical characteristics of suicide attempters

A comorbid psychiatric disorder was diagnosed among 50% of the suicide attempters (n=75), and substance use disorder (SUD) was noted among 23.3% of suicide attempters (n=35). The most common psychiatric comorbidity in the sample was depression (48.7%). High-intent suicide attempts were associated with the presence of psychiatric comorbidity (p=0.004) and an absence of SUD substance use (p=0.02). There was no association between mode of attempt, lethality of attempt, and psychiatric comorbidities.

While psychological distress was associated with comorbid psychiatric disorders, coping mechanisms, and resilience were found to be linked to SUD comorbidity: Subjective distress (p<0.001) and perceived stress scores (p<0.001) were higher, and resilience levels were lower (p=0.03) among attempters with comorbid psychiatric diagnoses than their counterparts. Compared to attempters without SUD, those with SUD comorbidity had greater emotion-focused coping (p=0.01), greater substance-mediated coping (p<0.001), and reduced resilience scores (p<0.05) (Table 4).

**Table 4:** Association between current suicide attempt, psychological factors, and clinical characteristics among suicide attempters (N=150) *p<0.05, statistically significant, **excluding substance use disorders CD-RISC: Connor Davidson Resilience Scale, GHQ: General Health Questionnaire, PSLES: Presumptive Stressful Life Events Scale.

Variable	Psychiatric comorbidity**		Test characteristic, p-value	Substance Use Disorder Comorbidity		Test characteristic, p-value
	Present (n=75) N (%) or Mean ± SD	Absent (n=75) N (%) or Mean ± SD		Present (n=35) N (%) or Mean ± SD	Absent (n=115) N (%) or Mean ± SD	
Current suicide attempt characteristics						
Mode of attempt						
Pesticide poisoning	32 (42.7)	38 (50.7)	χ^2 ^= 3.593 (0.32)	21 (60)	49 (42.6)	Fisher’s Exact = 5.437 (ns)
Tablet overdose	26 (34.7)	25 (33.3)		7 (20)	44 (38.3)	
Household poisons	8 (10.7)	9 (12.0)		3 (8.6)	14 (12.2)	
Hanging	9 (12.0)	3 (4.0)		4 (11.4)	8 (7)	
Suicide intent						
Low intent	16 (21.3)	34 (45.3)	χ^2 ^=11.191 (0.004)*	5 (14.3)	45 (39.1)	χ^2 ^= 7.872 (0.02)*
Medium intent	41 (54.7)	33 (44.0)		21 (60)	53 (46.1)	
High intent	18 (24.0)	8 (10.7)		9 (25.7)	17 (14.8)	
Lethality of current suicide attempt						
Risk score	7.17 ± 1.14	7.36 ± 1.42	t=-0.887 (0.37)	7.37 ± 1.44	7.23 ± 1.24	t=0.548 (0.58)
Rescue score	13.96 ± 1.46	13.95 ± 1.66	t=-0.052 (0.96)	13.83 ± 1.98	13.99 ± 1.41	t=-0.541 (0.59)
Risk-rescue score	29.13 ± 8.66	30.28 ± 10.49	t=-0.730 (0.47)	31.26 ± 12.10	29.23 ± 8.72	t=1.091 (0.28)
Psychological characteristics						
Psychological distress						
PSLES total	149.20 ± 69.65	176.41 ± 92.19	t=-2.040 (0.40)	163.69 ± 85.11	162.54 ± 82.15	t=0.072 (0.94)
Perceived stress scale score	27.69 ± 3.61	20.19 ± 6.36	t=8.883 (<0.001)*	25.37 ± 5.74	23.50 ± 6.53	t=1.521 (0.13)
GHQ scale score	13.52 ± 5.09	9.76 ± 3.85	t=5.097 (<0.001)*	12.03 ± 4.10	11.52 ± 5.11	t=0.537 (0.59)
Coping strategies						
Problem-focused coping	50.19 ± 7.28	51.04 ± 7.67	t=-0.699 (0.48)	48.74 ± 6.55	51.18 ± 7.66	t=-1.704 (0.09)
Emotion-focused coping	49.83 ± 7.58	51.27 ± 8.07	t=-1.127 (0.26)	47.31 ± 6.66	51.53 ± 7.94	t=-2.854 (0.01)*
Less useful coping	33.49 ± 4.36	32.83 ± 5.29	t=0.842 (0.40)	32.09 ± 4.72	33.49 ± 4.85	t=-1.506 (0.13)
Substance use coping	6.32 ± 4.46	6.19 ± 4.45	t=0.183 (0.85)	13.66 ± 3.59	0	t=29.803 (0.001)*
Humor	4.32 ± 1.42	4.17 ± 0.86	t=0.763 (0.45)	4.06 ± 0.34	4.30 ± 1.33	t=-1.090 (0.28)
Resilience						
CD-RISC score	69.51 ± 12.71	73.76 ± 11.68	t=-2.134 (0.03)*	75.17 ± 10.65	70.56 ± 12.67	t=1.954 (0.05)*

Psychological characteristics and suicidal behavior

Association Between Psychological Characteristics and Past Suicide Attempt

The associations between psychological characteristics (life event severity, perceived stress, and subjective distress), protective mechanisms (coping and resilience), and past suicide attempt were explored. The results revealed increased subjective distress, diminished problem-focused coping mechanisms, and reduced resilience levels among those with past suicide attempt than those without such a history (Table 5).

**Table 5:** Association between psychological characteristics (risk factors, coping strategies, and resilience) and history of past suicide attempts *p<0.05, statistically significant CD-RISC: Connor Davidson Resilience Scale, GHQ: General Health Questionnaire, PSLES: Presumptive Stressful Life Events Scale.

Variables	Past suicide attempt		Test characteristic, p-value
	Present (n=35) Mean ± SD	Absent (n=115) Mean ± SD	
Psychological distress			
PSLES total	142.31 ± 62.72	169.04 ± 86.99	t=-1.687 (0.09)
Perceived stress scale score	25.23 ± 5.85	23.55 ± 6.52	t=1.367 (0.18)
GHQ scale score	13.03 ± 4.91	11.22 ± 4.82	t=1.940 (0.05)*
Coping strategies			
Problem-focused coping	48.66 ± 5.87	51.21 ± 7.81	t=-2.074 (0.04)*
Emotion-focused coping	49.00 ± 7.71	51.02 ± 7.84	t=-1.338 (0.18)
Less useful coping	33.74 ± 4.38	32.98 ± 4.97	t=0.813 (0.42)
Substance use coping	6.09 ± 4.26	6.30 ± 4.51	t=-0.54 (0.80)
Humor	4.17 ± 1.01	4.27 ± 1.22	t=-0.431 (0.67)
Resilience			
CD-RISC score	67.94 ± 10.81	72.76 ± 12.61	t=-2.040 (0.04)*

Association Between Psychological Characteristics and Current Suicide Attempt

Mode of suicide attempt: Perceived stress and subjective distress scores were the highest among those who attempted hanging. However, post hoc comparisons did not capture within-group differences. Though coping strategies were not linked to the mode of suicide attempt, resilience levels were significantly lower in those who attempted hanging than other modes (p<0.02) (Table 6).

**Table 6:** Association between psychological characteristics (risk factors, coping strategies, and resilience) and mode of current suicide attempt *p<0.05, statistically significant CD-RISC: Connor Davidson Resilience Scale, GHQ: General Health Questionnaire, PSLES: Presumptive Stressful Life Events Scale.

Variables	Mode of current suicide attempt				Test characteristic, p-value	Post hoc comparisons (Bonferroni method)
	Pesticide poisoning (A) (n=70) Mean ± SD	Tablet overdose (B) (n=51) Mean ± SD	Household poisons (C) (n=17) Mean ± SD	Hanging (D) (n=12) Mean ± SD		
Psychological distress						
PSLES total	171.36 ± 77.72	163.76 ± 85.39	142.47 ± 88.14	137.67 ± 90.56	F=0.967 (0.41)	NA
Perceived stress scale score	23.64 ± 5.87	23.53 ± 6.54	22.59 ± 6.71	29.33 ± 6.34	F=3.383 (0.02)*	ns
GHQ scale score	11.10 ± 4.44	12.08 ± 5.82	10.06 ± 3.13	15.17 ± 3.24	F=3.245 (0.02)*	ns
Coping strategies						
Problem-focused coping	49.96 ± 7.40	51.82 ± 6.48	51.47 ± 8.72	48.08 ± 9.61	F=1.165 (0.33)	NA
Emotion-focused coping	49.96 ± 7.83	52.67 ± 7.36	49.12 ± 5.81	47.00 ± 10.53	F=2.456 (0.06)	NA
Less useful coping	33.06 ± 4.71	33.18 ± 5.19	33.65 ± 3.26	33.00 ± 6.33	F=0.071 (0.97)	NA
Substance use coping	6.90 ± 4.98	5.33 ± 3.52	5.41 ± 3.37	7.58 ± 5.35	F=1.815 (0.14)	NA
Humor	4.23 ± 1.01	4.33 ± 1.54	4.24 ± 0.97	4.00 ± 0	F=0.271 (0.84)	NA
Resilience						
CD-RISC score	7.19 ± 1.27	7.53 ± 1.50	7.24 ± 0.83	6.67 ± 0.65	F=3.459 (0.02)*	D < A

Intent of suicide attempt

Suicide attempts with high intent to die were associated with the most significant subjective distress and perceived stress levels (p<0.001). In addition, high-intent attempts were associated with substance use coping (p=0.01) and reduced resilience levels than low-intent and medium-intent subgroups (p=0.03) (Table 7).

**Table 7:** Association between psychological characteristics (risk factors, coping strategies, and resilience) and intent of current suicide attempt *p<0.05, statistically significant,  CD-RISC-Connor Davidson Resilience Scale, GHQ-General Health Questionnaire, PSLES-Presumptive Stressful Life Events Scale

Variables	Intent of current suicide attempt			Test characteristic, p-value	Post hoc comparisons (Bonferroni method)
	Low intent (A) (n=50) Mean ± SD	Medium intent (B) (n=74) Mean ± SD	High intent (C) (n=26) Mean ± SD		
Psychological distress					
PSLES total	156.82 ± 93.35	169.91 ± 76.68	154.12 ± 77.97	F=0.546, p=0.58	NA
Perceived stress scale score	19.90 ± 6.91	24.77 ± 4.67	29.35 ± 4.56	F= 26.876, p<0.001*	C > B, C > A, B > A
GHQ scale score	8.98 ± 4.17	11.72 ± 3.75	16.54 ± 5.25	F= 27.945, p<0.001*	C > B, C > A, B > A
Coping strategies					
Problem-focused coping	52.66 ± 6.67	49.77 ± 7.27	49.08 ± 8.82	F= 2.978, p=0.05*	NS
Emotion-focused coping	52.50 ± 7.69	49.59 ± 7.69	49.50 ± 8.07	F=2.377, p=0.09	NA
Less useful coping	32.96 ± 5.14	33.53 ± 4.51	32.50 ± 5.26	F= 0.494, p=0.61	NA
Substance use coping	4.72 ± 2.61	6.86 ± 4.98	7.46 ± 4.96	F= 4.883, p=0.01*	C > A, B > A
Humor	4.52 ± 1.76	4.03 ± 0.23	4.35 ± 1.29	F= 2.803, p=0.06	NS
Resilience					
CD-RISC score	74.46 ± 12.10	71.54 ± 11.52	66.46 ± 13.06	F= 3.722, p=0.03*	C < A

Lethality of suicide attempt

Correlation analyses to find the relationship between lethality and psychological characteristics revealed that lethality of suicide attempt increased with increasing levels of resilience (p=0.02). No other relationships were observed between lethality and other psychological variables (Table 8).

**Table 8:** Association between psychological characteristics (distress, coping strategies, and resilience) and lethality of current suicide attempt *p<0.05, statistically significant,  CD-RISC-Connor Davidson Resilience Scale, GHQ-General Health Questionnaire, PSLES-Presumptive Stressful Life Events Scale.

Variables		Psychological distress (Pearson's *r*, p-value)			Coping strategies (Pearson's *r*, p-value)					Resilience (Pearson's *r*, p-value)	Lethality (Pearson's *r*, p-value)
		PSLES Total	Perceived Stress Score	GHQ total	Problem-focused coping	Emotion-focused coping	Less useful coping	Substance use coping	Humor coping	CD-RISC score	Risk-rescue score
Psychological distress	PSLES Total	-	-0.239 (0.003)*	0.012 (0.87)	0.106 (0.19)	0.089 (0.28)	-0.156 (0.06)	-0.011 (0.89)	-0.119 (0.15)	0.129 (0.12)	0.013 (0.87)
	Perceived Stress Score	-0.239 (0.003)*	-	0.603 (<0.001)*	-0.278 (<0.001)*	-0.276 (<0.001)*	0.178 (0.03)*	0.151 (0.06)	-0.017 (0.84)	-0.332 (<0.001)*	-0.053 (0.52)
	GHQ total	0.012 (0.87)	0.603 (<0.001)*	-	-0.121 (0.14)	-0.069 (0.40)	-0.052 (0.53)	0.057 (0.49)	0.049 (0.55)	-0.395 (<0.001)*	-0.038 (0.64)
Coping strategies	Problem-focused coping	0.106 (0.19)	-0.278 (<0.001)*	-0.121 (0.14)	-	0.769 (<0.001)*	-0.255 (0.002)*	-0.177 (0.03)*	0.188 (0.02)*	0.494 (<0.001)*	0.096 (0.24)
	Emotion-focused coping	0.089 (0.28)	-0.276 (<0.001)*	-0.069 (0.40)	0.769 (<0.001)*	-	-0.315 (<0.001)*	-0.254 (0.002)*	0.119 (0.15)	0.467 (<0.001)*	0.051 (0.53)
	Less useful coping	-0.156 (0.06)	0.178 (0.03)*	-0.052 (0.53)	-0.255 (0.002)*	-0.315 (<0.001)*	-	-0.104 (0.20)	-0.085 (0.30)	-0.157 (0.05)*	0.081 (0.19)
	Substance use coping	-0.011 (0.89)	0.151 (0.06)	0.057 (0.49)	-0.177 (0.03)*	-0.254 (0.002)*	-0.104 (0.20)	-	-0.092 (0.26)	0.116 (0.16)	0.054 (0.52)
	Humor	-0.119 (0.15)	-0.017 (0.84)	0.049 (0.55)	0.188 (0.02)*	0.119 (0.15)	-0.085 (0.30)	-0.092 (0.26)	-	-0.014 (0.08)	-0.079 (0.34)
Resilience	CD-RISC score	0.129 (0.12)	-0.332 (<0.01)*	-0.395 (<0.01)*	0.494 (<0.001)*	0.467 (<0.001)*	-0.157 (0.05)*	0.116 (0.16)	-0.014 (0.08)	-	0.193 (0.02)*
Lethality	Risk-rescue score	0.013 (0.87)	-0.053 (0.52)	-0.038 (0.64)	0.096 (0.24)	0.051 (0.53)	0.081 (0.19)	0.054 (0.52)	-0.079 (0.34)	0.193 (0.02)*	-

Inter-relationship among various psychological characteristics

While the perceived stress score increased with increasing levels of subjective distress (<0.001), it shared an inverse relationship with life event severity (p=0.003). Increased perceived stress was associated with an increased likelihood of less useful coping (p=0.03) and reduced likelihood of problem- or emotion-focused coping mechanisms (p<0.001).

Among the various coping mechanisms, the problem-focused coping mechanism was positively correlated with emotion-focused (p<0.001) and humor coping strategies (p=0.02), whereas the same had a negative relationship with less useful coping (p=0.002) and substance use coping mechanisms (p=0.03).

Increased resilience was associated with reduced perceived stress scores (p<0.001) and reduced subjective distress (p<0.001). In addition, higher levels of resilience positively correlated with problem-focused (p<0.001) and emotion-focused coping strategies (p<0.001). Negative correlation was observed between resilience and less useful coping strategies (p=0.05) (Table 8).

## Discussion

The present study explored the relationships existing between psychological distress, protective mechanisms (coping and resilience), and suicidal behavior profile among persons with a history of suicide attempts in the past month.

Sociodemographic characteristics and gender differences

The study sample’s gender distribution and other demographic characteristics are similar to previous studies from this part of the country [35,59]. The prevalence rates of past history and family history of suicide attempts are comparable with previous studies from this region [49,60].

The differences in the mode of suicide attempt (tablet overdose among females and poisoning among males) are similar to previous studies [49] and could be explained by the gender differences in personality characteristics, sociocultural reasons, and access to means of suicide attempt [61,62]. The lack of gender differences based on the lethality of suicide attempts in our study reiterates the findings of recent reviews of suicide-related deaths in India [63,64].

As noted in previous studies, we found that female attempters employed emotion-focused problem-solving approaches, and males preferred substance use as a coping strategy [65,66]. We found comparatively lesser degrees of resilience among females than males, probably due to the effects of gender roles, psychosocial hardships, and sociocultural pressure among women than men [67].

Current suicidal behavior

Though national data report that hanging is the most common method of suicide attempt [2], poisoning was the most common type of suicide attempt among the study sample, which is similar to previous studies from this region that reported consumption of pesticide poisons outweighed hanging as the most common mode of suicide attempt [59,68]. This could possibly be due to agriculture being the predominant labor in this part of the country, which could provide easy access to pesticides [23,69,70]. The majority of our sample with impulsive, low- or medium-intent suicide attempts suggests that impulsivity and related traits might accelerate the suicidal process among attempters [48,71].

Clinical factors of suicide attempters

Despite the impulsive nature of most suicide attempts, our findings reiterate that depression remains the most common comorbid psychiatric disorder associated with a recent suicide attempt [72,73]. The findings also support that psychiatric comorbidity is associated with increased stress levels and more severe suicide attempts [73]. However, the link between high-intent suicide attempts and lack of substance abuse needs further exploration.

Psychological characteristics among suicide attempters

Psychological Distress

The positive association between perceived stress and psychiatric comorbidities is similar to those reported in previous studies [74,75]. A past history of suicide attempts was associated with increased stress levels as noted in a systematic review comparing multiple attempters vs. single attempters [76]. In contrast with the existing literature, we did not find an association between stressful life events severity and past suicide attempts [34,77,78]. This could be because of recall bias in participants’ recollection of SLEs. Though high-intent suicide attempts were less frequent in our study sample, such attempts were associated with increased subjective distress, which aligns with findings from previous studies [25].

Coping Mechanisms

As documented in the literature, emotion-focused coping was positively associated with SUD comorbidity [79,80]. The present study also revealed that past suicide attempt was associated with diminished problem-focused coping and added to the literature on unique aspects of repeat suicide attempters from this region [81]. Such findings could be explained by maladaptive cognitive mechanisms [82], including hopelessness [83], reduced social support [83], and personality characteristics [84] observed in multiple attempters.

Resilience

In our study, persons with hanging attempts had lower resilience when compared to other modes, indicating that resilience levels play a key role in mediating the seriousness of suicide attempt [38]. The negative relationship between resilience and psychiatric or SUD comorbidity is in line with previous studies, which revealed that resilience could be considered an “inverse” predictor of SUD [85].

Our findings reiterate that a past history of suicide attempt was associated with lower resilience levels [86]. Though depression could be considered a potential confounder, studies reveal that low resilience was independently associated with an increased risk of suicide across the lifespan [87,88]. The lower levels of resilience among the high-intent suicide attempters underscore the importance of resilience as a buffer in preventing suicide ideations and attempts [38].

Interestingly, our findings revealed that increased resilience was associated with more lethal suicide attempts. This contrasts with the literature stating that resilience is a protective factor against SB [12,38]. The difference could be explained by the fact that in our sample, male attempters had lower resilience and more substance use than females, both factors closely related to serious suicide attempts [89]. Increased resilience might also lead to better concealment of suicidal plans and feelings of increased shame, contributing to lethal suicide attempts. Future studies need to explore the complex dynamics between resilience and lethality of suicide attempt.

Interplay among the psychological characteristics

Interestingly, we found that though our sample encountered severe stressful life events, they experienced minimal distress, reflected by the inverse relationship between objective measures of life event severity and perceived stress levels. This could be due to chronic adaptation to psychological stress and developing positive coping strategies over time, leading to better handling of stressful life events [90]. Since low resilience levels were associated with multiple suicide attempts and high-intent suicide attempts in our sample, we further explored whether “resilience” interacted with the other psychological factors. In our sample, resilience had an inverse relationship with perceived stress and subjective distress, as noted in previous studies, which indicated that it acted as a buffer for experiencing stress [38].

Strengths and limitations

The present study adds to the dearth of Indian literature on psychological risk factors and protective mechanisms influencing suicidal behavior. It also increases the literature on the inter-relationship between such psychological characteristics. However, the generalizability of the present study’s findings is limited by the cross-sectional analysis of trait markers such as resilience and coping strategies, potential recall bias in data collection, selection bias in recruiting more impulsive attempters, and lack of data regarding early life adversities and personality characteristics. 

## Conclusions

Suicidal behavior is strongly associated with psychological risk factors and protective factors. Significant gender differences exist in the mode of suicide attempt, coping strategies, and resilience. Depression is the most common psychiatric comorbidity associated with suicide attempt. Increased perceived stress was related to the presence of psychiatric comorbidity, past history of suicide attempt, and high-intent suicide attempts. Maladaptive coping strategies were associated with substance abuse and the history of past suicide attempt. Low resilience levels were associated with hanging attempts, psychiatric or SUD comorbidity, past history of suicide attempt, high-intent suicide attempts, and less lethal suicide attempts.
